# Hearing loss in Africa: current genetic profile

**DOI:** 10.1007/s00439-021-02376-y

**Published:** 2021-10-05

**Authors:** Samuel Mawuli Adadey, Edmond Wonkam-Tingang, Elvis Twumasi Aboagye, Osbourne Quaye, Gordon A. Awandare, Ambroise Wonkam

**Affiliations:** 1 Department of Biochemistry, Cell and Molecular Biology, West African Centre for Cell Biology of Infectious Pathogens (WACCBIP), University of Ghana, P.O. Box LG 54, Legon, Accra, Ghana; 2 Division of Human Genetics, Faculty of Health Sciences, University of Cape Town, Anzio Road, Observatory, Cape Town, 7925 South Africa

## Abstract

Hearing impairment (HI) is highly heterogeneous with over 123 associated genes reported to date, mostly from studies among Europeans and Asians. Here, we performed a systematic review of literature on the genetic profile of HI in Africa. The study protocol was registered on PROSPERO, International Prospective Register of Systematic Reviews with the registration number “CRD42021240852”. Literature search was conducted on PubMed, Scopus, Africa-Wide Information, and Web of Science databases. A total of 89 full-text records was selected and retrieved for data extraction and analyses. We found reports from only 17/54 (31.5%) African countries. The majority (61/89; 68.5%) of articles were from North Africa, with few reports found from sub-Saharan Africa. The most common method used in these publications was targeted gene sequencing (*n* = 66/111; 59.5%), and only 13.5% (*n* = 15/111) used whole-exome sequencing. More than half of the studies were performed in families segregating HI (*n* = 51/89). *GJB2* was the most investigated gene, with *GJB2*: p.(R143W) founder variant only reported in Ghana, while *GJB2*: c.35delG was common in North African countries. Variants in *MYO15A* were the second frequently reported in both North and Central Africa, followed by *ATP6V1B1* only reported from North Africa. Usher syndrome was the main syndromic HI molecularly investigated, with variants in five genes reported: *USH2A*, *USH1G*, *USH1C*, *MYO7A*, and *PCDH15*. *MYO7A*: p.(P1780S) founder variant was reported as the common Usher syndrome variant among Black South Africans. This review provides the most comprehensive data on HI gene variants in the largely under-investigated African populations. Future exomes studies particularly in multiplex families will likely provide opportunities for the discovery of the next sets of novel HI genes, and well as unreported variants in known genes to further our understanding of HI pathobiology, globally.

**Supplementary Information:**

The online version contains supplementary material available at 10.1007/s00439-021-02376-y.

## Introduction

Hearing impairment (HI) is the most prevalent sensory disability with a growing concern globally (Mulwafu et al. 2016; Olusanya et al. 2014). The World Health Organisation’s (WHO) estimates of the number of people living with disabling HI globally increased from 278 million in 2005, to 360 million in 2012, and 466 million in 2018 (6.1% of the world population) (Mulwafu et al. 2016). Based on the WHO projections, 2.5 billion people would have some form of HI by 2050. HI is higher in sub-Saharan Africa, as it occurs in 6 per 1000 live births as compared to a lower incidence of 1 per 1000 live births in high-income countries (Olusanya et al. 2014). Depending on its pathophysiology, HI can be conductive (resulting from abnormalities of the external ear and/or the ossicles of the middle ear), sensorineural (results from a malfunction of inner ear structures), or mixed (a combination of conductive and sensorineural HI) (Shearer et al. 2017). The etiologies of HI vary from genetic to environmental factors, while some HI cases have an unknown cause (Wonkam Tingang et al. 2020a; Wonkam et al. 2019). Genetic factors contribute to 30–50% of HI cases of childhood HI in sub-Saharan Africa (Lebeko et al. 2015). HI of genetic origin may be syndromic, where there are other clinical features associated with the loss of hearing. Conversely, HI may be non-syndromic whereby HI is the only observed clinical feature (Lebeko et al. 2015).

Syndromic HI accounts for up to 30% of hereditary HI and over 400 syndromes associated with HI have been described to date, including Waardenburg syndrome, Branchiootorenal syndrome, Usher syndrome, Pendred syndrome, Keratitis–ichthyosis–deafness syndrome, and Alport syndrome (Bayazit and Yılmaz 2006; Lebeko et al. 2015; Shearer et al. 2017). Non-syndromic HI (NSHI) accounts for approximately 70% of all hereditary Hl cases (Bayazit and Yılmaz 2006; Shearer et al. 2017). The inheritance pattern among neonates with NSHI is approximately 80% inherited as autosomal recessive, about 20% autosomal dominant, and < 1% X-linked or mitochondrial (Shearer et al. 2017; Smith et al. 2005). NSHI is highly genetically heterogeneous, with approximately 170 loci and 123 genes identified to date (Van Camp and Smith 2006). The most common variants associated with autosomal recessive non-syndromic HI (ARNSHI) have been found within the connexin genes, and they have been prevalent among European, and Asian populations (Lebeko et al. 2015). Connexins were shown to be expressed in the inner ear, and some studies support their role in the metabolism of potassium and nutrient in the cochlea (Adadey et al. 2020b; Xu and Nicholson 2013).

*GJB2* (on chromosome 13q12) is the most common connexin gene associated with ARNSHL in European and Asian populations accounting for almost 50% of cases (Adadey et al. 2020b; Bayazit and Yılmaz 2006). The most common *GJB2* variant is c.35delG which is seen in up to 70% of cases. *GJB2* c.35delG is prevalent throughout Europe, North Africa, and the Middle East, as well as areas populated largely by immigrants from these regions (Bayazit and Yılmaz 2006; Lebeko et al. 2015). Other *GJB2* variants are prevalent in specific populations, including c.235delC among Asians, p.W24X in Indians, 167delT among Ashkenazi Jews, and p.R143W in Africans from Ghana (Adadey et al. 2020b; Brobby et al. 1998; Chan and Chang 2014). Apart from connexin genes, other common genes implicated in HI among European and Asian populations include *SLC26A4* (implicated in Cochlear Homeostasis), *MYO15A* (involved in Cellular Organization), *OTOF* (involved in Neural transmission), *TMC1*, *CDH23* (implicated in Cellular Organization), and *TMPRSS3* (Duman and Tekin 2012).

The patterns of variations in African populations, the much diverse in the world, are shaped by ancestry reasons with Africans being the oldest human population, intra-African and out and back to Africa migration dynamics associated with population admixture, and ecological reasons with the north–south axis of the African continent that is associated with drastic differences in climate, diet, and exposure to infectious disease, all of which are motor of natural genomic selections (Wonkam 2021). Genetic variations of African populations have suggested three major divisions of the continent: (1) Mediterranean North Africa, (2) sub-Saharan Middle, West, and East Africa, and (3) southern regions of Africa (Campbell and Tishkoff 2008; Reed and Tishkoff 2006). Moreover, population genetic analysis using geographically, and ethnically diverse Africans indicated that there are over 13 genetically distinct populations and high levels of population admixture in Africa (Campbell and Tishkoff 2008), which will favour the much-needed discovery of new HI genes in order to improve diagnosis and care in Africa and globally (Chakchouk et al. 2019; Lebeko et al. 2016, 2017).

Although there are HI genetic studies from Africa, with the majority from the Mediterranean north African populations, the genetic etiology of HI in most African populations remains elusive. Indeed, apart from the report in Ghana, pathogenic or likely pathogenic (PLP) variants in *GJB2* do not seem to contribute much to NSHI in most sub-Sahara African populations (Wonkam Tingang et al. 2020). Besides, targeted sequencing panels (with over 100 HI genes) have detected a consistently lower rate of pathogenic and likely pathogenic (PLP) variants in sporadic HI cases of African ancestry e.g. African Americans (26%), and Nigerians and Black South Africans (4%), compared to > 70% for Europeans and Asians (Sloan-Heggen et al. 2016; Yan et al. 2016). Therefore, there is an urgent need to investigate the genetic etiologies of HI across Africa, to improve our knowledge of variants and genes that contribute to HI in these populations. The present review provides the current state of knowledge on the genetics of hereditary HI in Africa.

## Methods

### Search strategy and data extraction

**Fig. 1:**
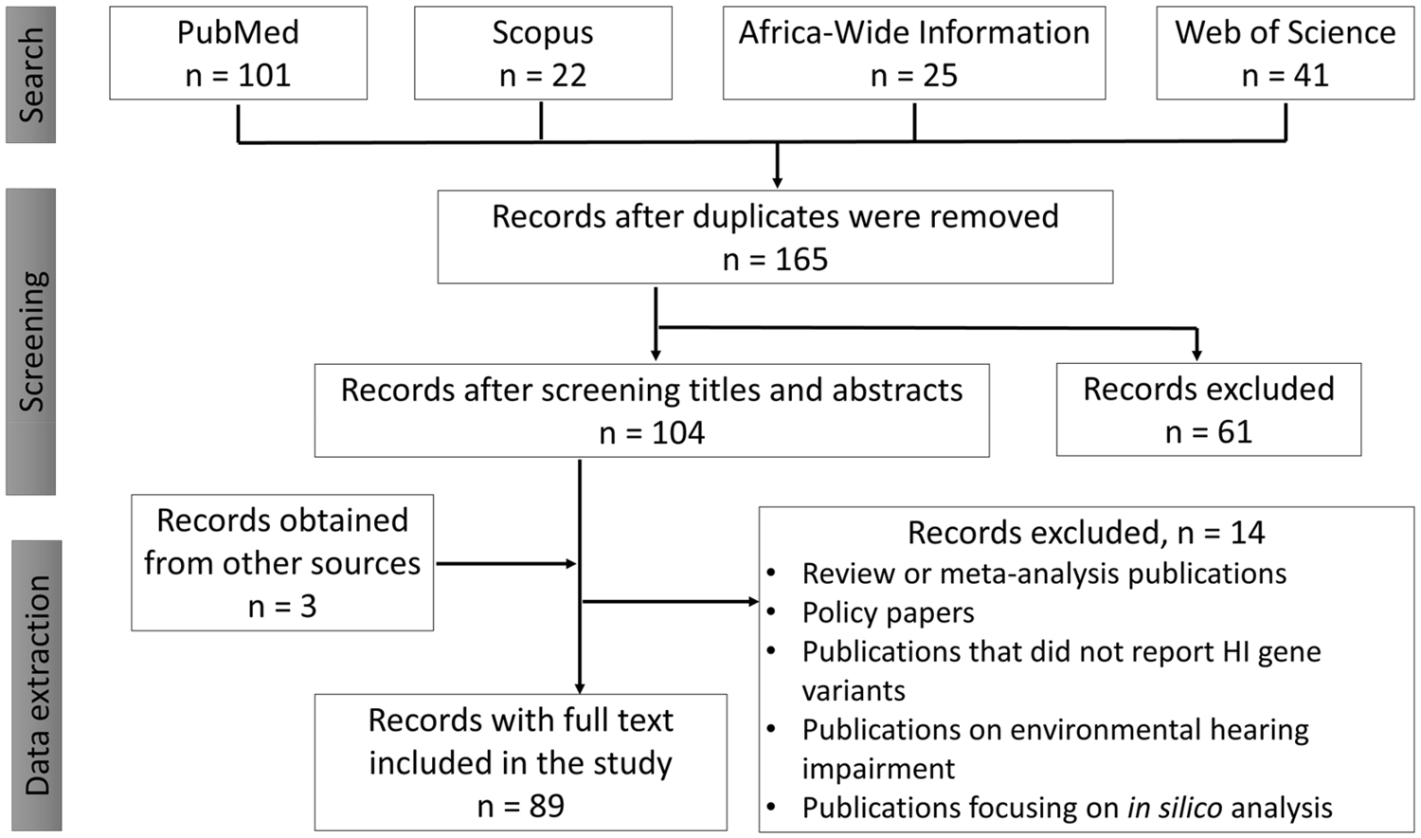
Flow diagram showing the screening process of records

The keywords: hearing impairment, genetics/genomics, diagnosis, and Africa were used to develop the search term “Hearing Impairment” OR “Hearing loss” OR “Deafness”) AND (“Genetics” OR “Genomics”) AND (“Testing” OR “Screening” OR “Diagnosis”) AND “Africa”. The search was conducted by two independent reviewers on PubMed, Scopus, Africa-Wide Information, and Web of Science databases (Fig. 1). The search was conducted between 1st to 14th March 2021. We registered the protocol on PROSPERO, International Prospective Register of Systematic Reviews with the registration number “CRD42021240852”.

To remove any form of bias, the data extraction was conducted by two independent reviewers (SMA and ETW) using structured data extraction Microsoft Excel spreadsheets (Office 365 education license under the University of Cape Town, South Africa). Based on the inclusion/exclusion criteria outlined below, data was extracted from 89 full-length publications out of 182 screened. The data extraction form consisted of the following data points (1) name of first and corresponding authors, (2) date of publication, (3) population investigated (4) total number of affected people investigated, (5) gene under investigation, (6) number of reported mutant alleles per variant. An expert in the field of genetics and HI (AW) was consulted whenever there was a disagreement between the judgment of the two reviewers. The extracted data were imported into SPSS version 25 (IBM, Armonk, New York, United States) and analyzed.

#### Inclusion criteria

- Publications from Africa on hearing impairment
- Publications on genetics/genomics of hearing impairment
- Full-length articles.

#### Exclusion criteria

- Review or meta-analysis publications
- Policy papers
- Publications that did not report HI gene variants
- Publications on environmental hearing impairment
- Publications focusing on in silico analysis.

### Quality assessment

The quality of the documents reviewed was assessed by independent reviewers (SMA and EWT) prior to data extraction. The quality was assessment was conducted using the quality of genetic studies (Q-Genie) tool developed by Sohani et al. (2016). The tool designed by Hoy et al. (2012) was used to assess the risk of bias for the prevalence studies. By discussion and consensus, and sometimes with the consultation of an expert in the field (AW), discrepancies and differences in the judgment of the reviewers (SMA and EWT) were resolved.

### Clinical significance assessment of variants

The clinical significance was assessed for the identified variants on 3 variant databases; InterVar (Li and Wang 2017), VarSome (Kopanos et al. 2019), and ClinVar (Landrum et al. 2020). VarSome and InterVar were tools used for assessing the clinical significance of variants based on the American College of Medical Genetics and Genomics (ACMG)/Association for Molecular Pathology (AMP) 2015 guidelines (Li and Wang 2017). In addition, ClinVar Strong was used to provide further evidence of the clinical significance of the variants.

## Results

A total of 189 records were retrieved from the various databases searched, 25 retrieved from Africa Wide Information, 101 from PubMed, 22 from Scopus, and 41 from Web of Science. The retrieved records were combined, and duplicates were removed. The titles of the retrieved documents were used for the first level screening followed by the abstracts and 89 records were included in the study (Fig. 1). The 89 publications considered for the review dated from 1998 to 2021 with, a slowly increasing trend in publication numbers (Figure S1A). The geographical representation of published records (Figure S1B) showed that most HI studies were conducted in Northern Africa (61/89; 68.5%), with Tunisia accounting for a third of publications (28/89; 31.5%).

Considering families with two or more affected members as familial cases, we observed that a slightly higher number was recorded for publications that focused on familial cases (41; 46.1% publications), compared to non-familial cases (36 publications, 40.5%) (Figure S2A). Ten (11.2%) publications report on both familial and non-familial cases while two (2.2%) publications investigated HI genes in hearing controls. The majority of familial studies reported on one to five familial cases (20 publications), while 13 publications reported on 51–99 familial cases (Figure S2B). Only 5 publications reported on more than 99 familial cases. Northern African countries were found to report the highest percentage of consanguineous families with Tunisia having the highest number of publications (22/89 studies) that reported on consanguineous families (Figure S1B).

### Hearing impairment genes in Africa

**Fig. 2:**
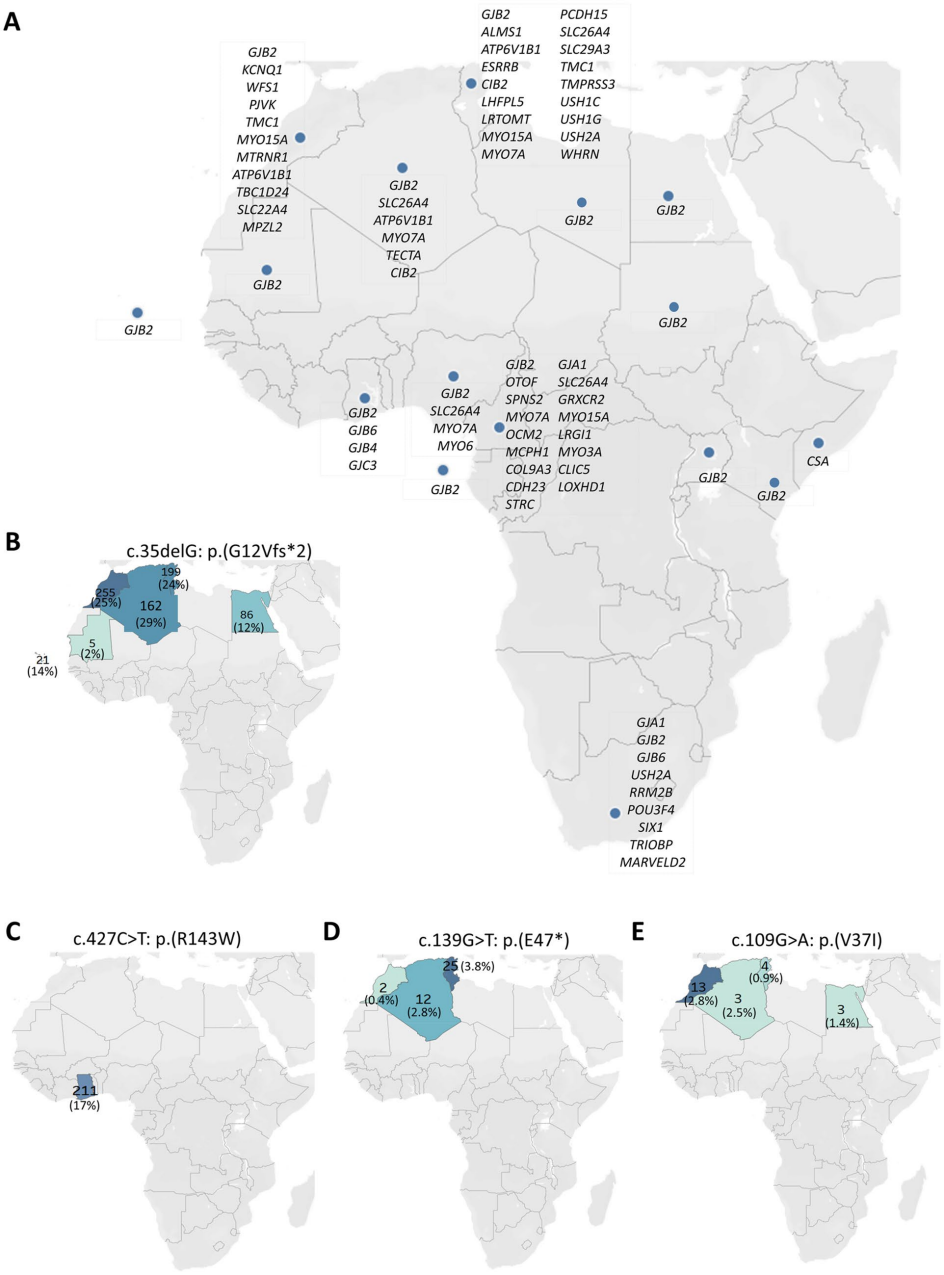
Geographical distribution of HI genes in Africa. **A** A geographical plot of the genes reported from different African countries. The geographical distribution of four common *GJB2* variants reported in Africa: **B** *GJB2*: c.35delG: p.(G12Vfs*2), **C** *GJB2*: c.427C>T: p.(R143W), **D** *GJB2*: c.139G>T: p.(E47*), **E** *GJB2*: c.109G>A: p.(V37I). The total number of mutant alleles (allele frequency in percentages) per country was displayed on the map

The analysis of the retrieved data showed a heterogeneous nature of HI genes in Africa with 46 HI genes reported from 17 African countries (Fig. 2A), with most studies on *GJB2* which was reported in 76.5% (13/17) of the countries. From the studies conducted in Tunisia, the country with the highest number of publications, 18 HI genes were found. Cameroon, with 17 genes and emerging as the second country with a description of variants in numerous genes.

### Molecular methods reported

This review identified more than 15 molecular methods used to investigate HI gene variants. Targeted genes sequencing was the most used method in Africa (*n* = 66/111; 59.5%) followed by exome sequencing (*n* = 15/111; 13.5%). Only five studies combined exome sequencing techniques with targeted sequencing. Two studies used specifically designed North African Deafness Chip (Table S1). Nineteen out of the 89 studies (21.3%) were found to use combinations of more than one method to screen for HI genes, which mostly consisted of restriction fragment length polymorphism (RFLP)-PCR combined with Sanger sequencing or targeted sequencing combined with exome sequencing. None of the studies reviewed used whole-genome sequencing (Table S1). It is worth noting that the studies found in some countries (Kenya, Mauritania, São Tomé and Príncipe, Somalia, Sudan, and Uganda) used only targeted sequencing which is effective but not comprehensive (Table S1).

### Connexin genes

#### Connexin 26 (*GJB2*) variants

We identified 28 PLP variants in *GJB2* gene (Table S2), of which c.35delG was the most associated variant (Table S3). The top four *GJB2* variants (variants with more than 20 reported alleles) were found to be c.35delG (728/3544 alleles, 20.5%), p.(R143W) (211/1230 alleles, 17.2%), p.(E47*) (39/1552 alleles, 2.5%), and p.(V37I) (23/1260 alleles, 1.8%). The rest 22 *GJB2* variants had less than 20 reported alleles (Table S3). *GJB2*: c.35delG: p.(G12Vfs*2) was reported only in the Northern African countries with most cases from Morocco. Similarly, *GJB2*: c.139G>T: p.(E47*) and c.109G>A: p.(V37I) variants were common in North African countries but not sub-Saharan African countries. Ghana was the only African country with a report of high-frequency *GJB2*: c.427C>T: p.(R143W) founder variant (Fig. 2). There were studies from some sub-Saharan African countries such as Cameroon and South Africa that extensively investigated HI genes but did not record any significant contribution of *GJB2* variants to HI. One of such studies from Cameroon reported a variant of uncertain significance [*GJB2*: c.499G>A (p.V167M)] in the heterozygous state in an affected family (Wonkam et al. 2019).

#### *GJA1* and *GJB4* variants

*GJA1* (connexin 43) may not contribute to HI in Africa. However, benign and VUS variants were reported from South Africa and Cameroon (Table S4). In Ghana, seven variants in *GJB4* (connexin 30.3) were reported however, all except one were either benign or uncertain significance. A likely pathogenic *GJB4* variant [p.(N119T): c.356A>C] was reported in a non-familial case from Ghana (Table S4).

### Non-connexin NSHI gene variants

Except for connexin genes, 34 NSHI gene variants were identified from 7 countries: Algeria (Behlouli et al. 2016; Talbi et al. 2018), Cameroon (Lebeko et al. 2016; Wonkam-Tingang et al. 2020b, 2021; Wonkam et al. 2021a, b), Ghana (Adadey et al. 2021), Morocco (Amalou et al. 2021; Bakhchane et al. 2015a; Ebermann et al. 2007), Nigeria (Yan et al. 2016), South Africa (Yan et al. 2016), Tunisia (Souissi et al. 2021; Belguith et al. 2009; Bensaid et al. 2011; Chakchouk et al. 2015; Chiereghin et al. 2021; Tlili et al. 2005, 2008; Masmoudi et al. 2001). Tunisia recorded the highest number of NSHI genes with PLP variants (11/34). PLP variants in *MYO15A* were the most reported and accounted for 102 out of 420 (24.3%) mutant alleles (Table S5).

### Mitochondrial DNA variants

The analysis of the retrieved records has shown that mitochondria HI was mainly reported in three African sub-populations. Except for a few studies  from Cameroon (Trotta et al. 2011), Nigeria (Lasisi et al. 2014), and South Africa (Bardien et al. 2009); the remaining two reports were from North Africa (Table S6). The m.1555A>G variant was the most frequently associated mitochondrial variant found in two out of the three countries, Tunisia (Mkaouar-Rebai et al. 2010; Souissi et al. 2021) and Morocco (Nahili et al. 2010). However, the reported frequencies of m.1555A>G variant were less than 2%, indicating the low contribution of the variant to HI in the studies reviewed. Four (4) PLP mitochondrial RNA1 variants (MTRNR1: [1048C>T; 1462G>T, 1018G>A, 1503G>A]) were found within the Cameroonian population.

### Syndromic hearing impairment

Fourteen (14) publications from five African countries were identified to report on syndromic HI. Ten (10) syndromes were found, and Usher syndrome was the most frequently reported syndrome associated with HI, characterized at the molecular level.

#### Usher syndrome

Variants from three known Usher syndrome genes *USH2A*, *USH1G*, and *USH1C* were found from the records retrieved. However, *USH2A* was the most common gene with the highest number of alleles with PLP variants (Table S7). Usher syndrome type 2 was the common type of Usher syndrome found in this review. The patients with Usher syndrome had early onset of severe to profound HI and a post-pubertal onset of retinitis pigmentosa. Some Usher syndrome patients were found to carry mutations in *MYO7A*, and *PCDH15* genes. Among indigenous Black South Africans, *MYO7A* was identified as the major gene associated with HI, with p.(P1780S) founder variant as the most commonly associated variant (Kabahuma et al. 2021). The medical examination of *MYO7A* and *PCDH15* patients showed that they had Usher syndrome type 1. These patients reported profound congenital bilateral HI and childhood-onset retinitis pigmentosa resulting in a progressively constricted visual field and night blindness. Fundus examination revealed retinal degeneration in all the patients (Ben-Rebeh et al. 2016). Using WES, a novel *RRM2B* c.786G>T variant was identified, as a plausible disease-causing, in  two affected sibling of Afrikaner ancestry, in South Africa, presenting a recessive sensorineural hearing impairment, associated with rod-cone dystrophy and  kidney disease; the variant was replicated in two unrelated South African patients with similar clinical phenotype suggesting a founder effect  (Roberts et al. 2020).

#### Distal renal tubular acidosis

Distal renal tubular acidosis with HI was found to be associated with variants in *ATP6V1B1* gene with a total of 85 reported mutant alleles (*n* = 85/269; 31.6%). A high genetic and allelic heterogeneity was observed from the analysis of the reported variants, in that most variants were reported in only one study (Table S7). Three (3) out of the 50 variants, *ATP6V1B1*: c.1155dupC: p.(I386Hfs*56), *ATP6V1B1*: c.175-1G>C, and *TMC1*: c.100C>T: p.(R34X) were reported in 4, 3, and 2 different studies, respectively, the remaining variants were reported in one study only (Table S7). The analysis of retrieved genes associated with NSHI suggested that *ATP6V1B1* variants may be localized to only North Africa countries, suggesting a founder effect (Nagara et al. 2014). Variations in the *ATP6V1B1* gene were reported only from Algeria, Tunisia, and Morocco.

#### Alström syndrome

The Alström Syndrome patients belonged to a Tunisian family and *ALMS1*: c.10388-2A>G variant was identified as the likely cause of the condition (Chakroun et al. 2016). Two affected family members were examined in this family and found to have progressive vision and hearing loss, pendular nystagmus, and photophobia. Fundus examination of the affected individuals revealed sallow optic discs, attenuated vessels of the posterior poles, and pigment retinal degeneration. The patients had deep-set eyes and flat feet. Systemic/clinical examination did not identify other features such as hepatic dysfunction, abnormal digits, mental retardation, scoliosis, hypertension, renal dysfunction, alopecia, hypothyroidism, type 2 diabetes mellitus, hyperlipidemia, and acanthosis nigricans (Chakroun et al. 2016).

#### Cockayne syndrome

A novel splice site variant found in the Cockayne syndrome group A gene (ERCC8: c.551-1G>A) was associated with HI in two Somali siblings. The patients showed the features of Cockayne syndrome which included skin photosensitivity, progressive ataxia, spasticity, hearing loss, central and peripheral demyelination, and intracranial calcifications (Kleppa et al. 2007).

#### H syndrome

Five Tunisian unrelated patients were suspected to have the rare H syndrome with a characteristic phenotype of pigmentation and hypertrichosis patches. The other clinical features of H syndrome consisted of hepatosplenomegaly, hearing loss, heart abnormalities, and hypogonadism. Three *SLC29A3* variants [c.42delC: p.(S15Pfs*86), c.1088G>A: p.(R363Q), c.971C>T: p.(P324L)] were found to be associated with the condition (Jaouadi et al. 2018).

#### Jervell and Lange-Nielsen syndrome

Jervell and Lange-Nielsen syndrome was found in a Moroccan family that presented with congenital severe bilateral sensorineural HI. The affected patient had several episodes of syncope and was diagnosed with an associated *KCNQ1* (c.1343dupC, p.Glu449Argfs*14) variant (Adadi et al. 2017).

#### Pendred syndrome

A p.(L445W) variant in the Pendred syndrome (*PDS*) gene was associated with HI in Tunisian patients suspected to have Pendred syndrome. The affected patients reported sensorineural or mixed HI and an associated goiter in some cases. All the patients were found to have enlarged bilateral vestibular aqueducts, however, they had normal thyroid hormone levels (Charfeddine et al. 2010).

#### Keratitis–ichthyosis–deafness syndrome

An ectodermal defect that consists of an atypical ichthyosiform erythroderma linked to sensorineural deafness is referred to as Keratitis–ichthyosis–deafness (KID) syndrome (OMIM 148210). KID syndrome patients mostly have congenital HI. Two unrelated patients from Cameroon with KID syndrome were reported to have heterozygous *GJB2*-p.D50N mutation (Wonkam et al. 2013).

## Discussion

This review of literature focused on the landscape of genetic causes of HI in Africa, and it is the most comprehensive report, to date. The study suggested that a large majority of African countries are still to be investigated. The few available data using next-generation sequencing reveals expected genetic and allelic heterogeneity, and a high proportion of variants not previously reported (Bakhchane et al. 2015b; Ben-Rebeh et al. 2016; Oluwole et al. 2021; Wonkam et al. 2021b), that will definitively contribute to improving the disease-gene pair curation, globally. Moreover, there is evidence that novel HI genes will be discovered in Africa, based on the much lower pick-up rate when exploring patients with target genes panels (Lebeko et al. 2015; Yan et al. 2016).

Globally, connexins are the most associated genes to HI (Adadey et al. 2020b; Chan and Chang 2014); however, their contribution to HI in sub-Saharan Africa is negligible (Adadey et al. 2020b; Kabahuma et al. 2011; Wonkam 2015), except for Ghana (Adadey et al. 2019; Hamelmann et al. 2001) and North African (Abidi et al. 2008; Lucotte 2007) countries. Indeed, studies investigating the association of connexin gene variants with HI from other sub-Saharan African countries such as Cameroon (Bosch et al. 2014b; Tingang Wonkam et al. 2019), Nigeria (Lasisi et al. 2014), South Africa (Bosch et al. 2014b), Kenya, and Sudan (Gasmelseed et al. 2004) found a very low number of PLP variants in *GJB2*. The *GJB2*: 35delG is the most predominant variant in Europe (Adadey et al. 2020b; Gasparini et al. 2000), and the Mediterranean countries including Tunisia, Morocco, Egypt, and Algeria (Lucotte 2007). The *GJB2*: 35delG age was estimated at about 10,000–14,000 years ago, and emerged in the Mediterranean (Rothrock et al. 2003; Van Laer et al. 2001), and spread in Europe and Asia through the two Neolithic population movements (Dzhemileva et al. 2010). The other *GJB2* PLP variants identified in North Africa were *GJB2*: c.139G>T: p.(E47*) and c.109G>A: p.(V37I). The *GJB2*: c.109G>A: p.(V37I) is the most common in Asia, particularly in China (Adadey et al. 2020b). The *GJB2*: c.427C>T: p.(R143W) variant is the most prevalent variant associated with HI among Ghanaians (Adadey et al. 2019, 2020a; Brobby et al. 1998; Hamelmann et al. 2001), and was described as a founder variant in Adamorobe (Brobby et al. 1998), a village in the Eastern Region of Ghana. The carrier frequency of the variant among the hearing Ghanaian population was estimated at 1.4% (Adadey et al. 2019). The *GJB2*: c.427C>T: p.(R143W) variant is not exclusive to Ghana and has been found among Americans, likely through the slave trade, and in some Asian populations (Adadey et al. 2020b). Haplotype analysis of hearing-impaired individuals from the Japanese population suggested that the *GJB2*: c.427C>T: p.(R143W) founder variant occurred as multiple events (Shinagawa et al. 2020). Additional studies in Ghana to estimate the age of this variant is needed.

The current review suggested that non-*GJB2* connexins genes do not contribute significantly to HI in Africa. Though studies from South Africa (Bosch et al. 2014a) and Ghana (Adadey et al. 2020a) reported variants in *GJA1* and *GJB4*, almost all the variants were predicted as benign or uncertain significance. A likely pathogenic *GJB4* variant was found in a hearing-impaired participant from Ghana (Adadey et al. 2020a). GJB4 protein oligomerizes with other connexins in the cells of the inner ear to form gap junctions for the transport of ions across the cell. The expression of the gene in mouse (Bult et al. 2019) and rat (Wang et al. 2010) cochlea provides evidence of its importance in the auditory system. There is a need to sample many other African populations to understand the contribution of the *GJB4* to HI.

*MYO15A* and *ATP6V1B1* genes were the most common non-*GJB2* genes with PLP in Africa. *MYO15A* is an autosomal recessive deafness gene located on the human chromosome 17p11.2. An animal study has shown that the gene is expressed in the inner ear of mice (Kanzaki et al. 2006). It was found in mice that there is an interaction of the C-terminal PDZ domains of MYO15A and WHRN for recruiting endogenous WHRN to the tip stereocilia of hair cells (Belyantseva et al. 2005). *ATP6V1B1* is located on chromosome 2p13.3 of the human genome. According to the Online Mendelian Inheritance in Man (OMIM) database, *ATP6V1B1* associates with distal renal tubular acidosis 2 with progressive sensorineural hearing loss (MIM 267300) as syndromic phenotype. Studies from Africa reported *ATP6V1B1* association with distal renal tubular acidosis patients with sensorineural HI (Boualla et al. 2016; Dahmani et al. 2020; Elhayek et al. 2013). Unlike *MYO15A* PLP variants that were found in Central and North African countries, *ATP6V1B1* variants were reported only in North African countries from Algeria (Dahmani et al. 2020), Tunisia (Elhayek et al. 2013), and Morocco (Boualla et al. 2016). The localization of *ATP6V1B1* variants in North African countries may be due to a founder effect. In addition, the high prevalence of consanguinity within North African populations (El Bouchikhi et al. 2020; Mete et al. 2020) may be the driving force for the spread of *ATP6V1B1* variants in these populations. However, the association of *ATP6V1B1* variants with HI is not exclusive to North Africa, there were reports from other parts of the world including the USA (Subasioglu Uzak et al. 2013), and Japan (Yashima et al. 2010).

Mitochondrial gene variants have been associated with different multisystem syndromes including the nervous system, neuromuscular, and endocrine organs (Finsterer 2020; Mkaouar-Rebai et al. 2013a). We found seven studies from Cameroon (Trotta et al. 2011), Morocco (Nahili et al. 2010), and Tunisia (Mkaouar-Rebai et al. 2006, 2013a, b; Tabebi et al. 2015) that reported mitochondria variants in HI. The mitochondria are known to generate free radicals and reactive oxygen species (ROS) which may result in acoustic trauma when they accumulate in the cochlea (Le Prell et al. 2007; Yan and Liu 2010). Most mitochondria NSHI are caused by mutations in the 12S ribosomal RNA and tRNASer (UCN) genes which are maternally inherited (Guan 2004). Our review identified 12S rRNA m.1555A>G mutation as the most prevalent mitochondria variant associated with NSHI. Variable phenotypes such as the age of onset and severity have been reported in patients with the 12S rRNA m.1555A>G variant (Friedman et al. 1999). The phenotypic variability of mitochondria-associated HI is also sometimes influenced by *GJB2* mutations (Yan and Liu 2010). It is therefore important to investigate mitochondria variants in Ghanaian and North African populations towards the understanding and identification of HI gene modifiers in Africa. Owing to the diversity of the African population, it is important to study mitochondria variants in different African populations to understand their contribution to the development of HI.

The outcome of our review showed that Usher syndrome was the most common syndromic HI studied at the molecular level in Africa. Usher syndrome is characterized by HI and retinitis pigmentosa. Damage to the inner ear that impedes the inner ear functioning is the possible cause of HI and balance problems in Usher syndrome patients (Möller et al. 1989). According to phenotypic expressions (severity of hearing impairment, age of retinitis pigmentosa onset, and the presence or absence of vestibular response), there are three main types of Usher syndrome, and type 1 is the most severe form (Jaijo et al. 2007). Usher syndrome type 2 was found to be the most common type of Usher syndrome in Africa. Similar to the African observation, type 2 was reported as the main type of the syndrome in Canada (Ebermann et al. 2009). Five genes (*USH2A*, *USH1G*, *USH1C*, MYO*7A*, and *PCDH15*) were associated with Usher syndrome from Africa. A recent study among Black South Africans identified *MYO7A* as the most prevalent gene associated with syndromic HI in the South African population and the founder variant p.(P1780S) was the most implicated variant (Kabahuma et al. 2021).

### Expert’s comment and perspectives

Despite the great value provided by this review, there are indications that the results do not represent the full extent of HI genes in Africa. First, the genetics of HI is yet to be studied in most sub-Saharan African countries. Second, some studies excluded reported on known syndromic conditions with HI and did not investigate them at the molecular level. Indeed there are clinical reports suggesting that Waardenburg syndrome is the most common syndromic HI in numerous sub-Saharan African populations (Adadey et al. 2019; Tingang Wonkam et al. 2019); however, no report on the genetics of this condition was found in the literature. Third, less than 20% of studies used next-generation sequencing (with none using Whole-Genome Sequencing), which means that a fair number of genes and variants have not been investigated, owing to the high genetic and allelic heterogeneity of HI. Finally, almost all the studies reviewed focused on childhood HI (except for two), meaning that genes associated with adult HI still need to be uncovered in Africa. Therefore, the data provided by the present review have some limitations in its usability to design and develop a single diagnostic approach for screening HI in all parts of the continent. To address this challenge: there is a need to systematically investigate more African populations, using multiplex families, and next-generation sequencing (WES and WGS) to identify novel genes and their associated variants towards the development of population-specific diagnostic approaches. This would be facilitated by a high fertility rate in most of Africa, and a high consanguinity rate in some parts of Africa.

## Conclusion

This review provides the most comprehensive data on HI gene variants and emphasizes that most African populations are largely under-investigated, with reports only found in nearly a third of African countries. *GJB2* appeared to be the most investigated HI gene on the continent, yet its contribution to the burden of the disease was negligible in most sub-Saharan African populations, but for those from North Africa where *GJB2*- 35delG, p.(E47*) and p.(V37I) were predominantly found, and Ghana where high frequency of the *GJB2*-p.(R143W) founder variant was reported. *MYO15A* was the second frequently reported gene associated with NSHI in both North and Central Africa. *ATP6V1B1* variants were associated with distal renal tubular acidosis patients with sensorineural HI and only reported from North Africa. Usher syndrome was the most common syndromic HI genetically investigated.

The poor investigation of the genetics of HI in most African populations, and the limited use of WES for the available data suggested that the current report is likely an underestimation of the real spectrum of genes and variants associated with HI among Africans, considering the high heterogeneity of HI and the genetic diversity in Africa. The present review provides evidence that African populations are important in the discovery of the next sets of novel HI genes, this is further favored by the high fertility and consanguinity rates in some Africa regions and will contribute to improving our understanding of HI pathobiology, globally.

## Supplementary Information

Below is the link to the electronic supplementary material.

Supplementary file1 (DOCX 4733 kb)
