# Supplementary material for: Hearing loss in Africa: current genetic profile
Source: Hum Genet. 2021 Oct 5;141(3-4):505–17. doi: 10.1007/s00439-021-02376-y (PMC9034983; doi:10.1007/s00439-021-02376-y)
Supplement: Supplementary file 1 — Supplementary file1 (DOCX 4733 kb) [file 439_2021_2376_MOESM1_ESM.docx]

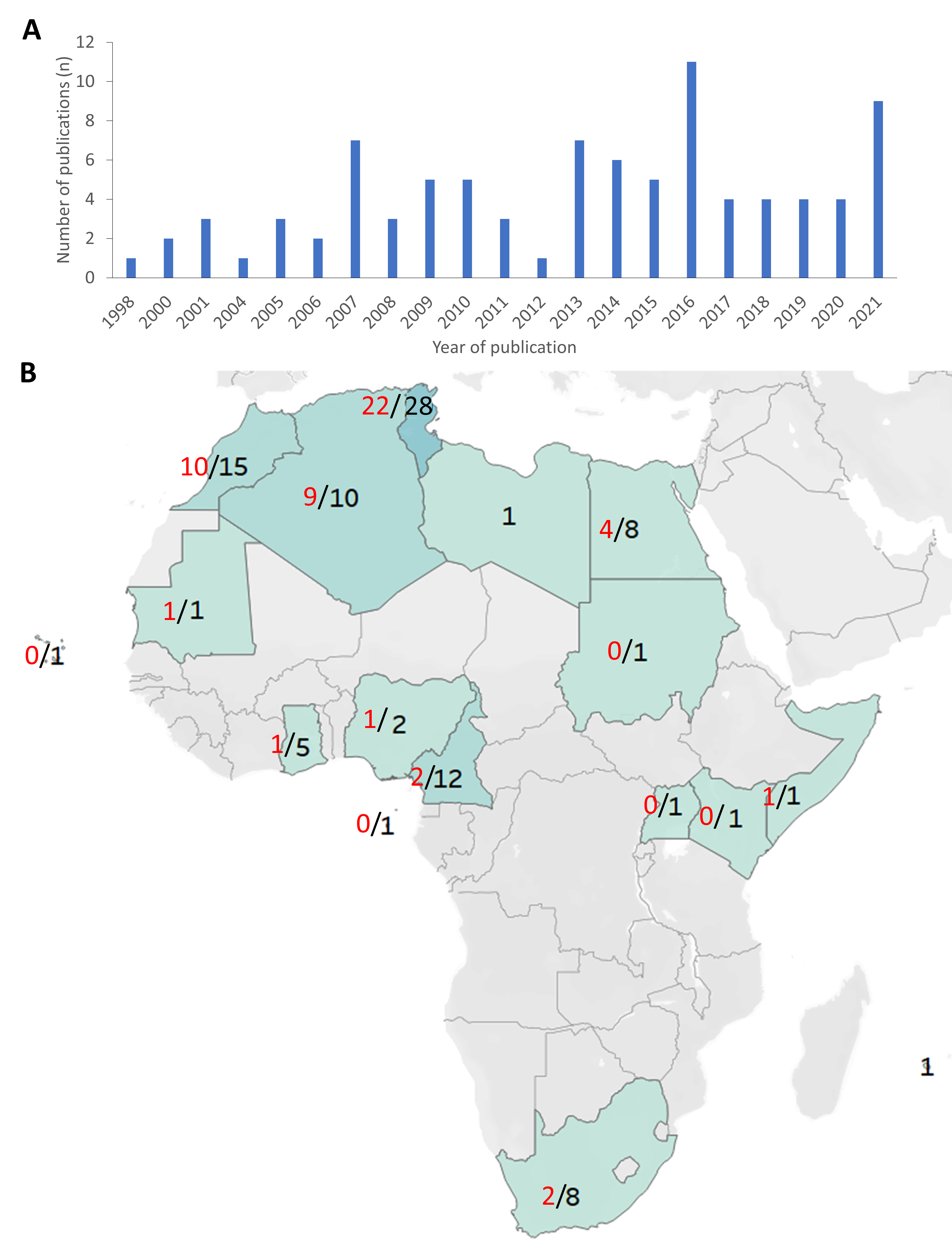


Figure S1: Year of publication and geographical presentation of retrieved records. (A) A plot of the year of publication of the records included in the study. (B) geographical representation of the study populations retrieved from the records used. The numerator (numbers in red) denotes the number of studies that reported consanguineous families and the denominator (numbers in black) represents the total number of studies from the country.


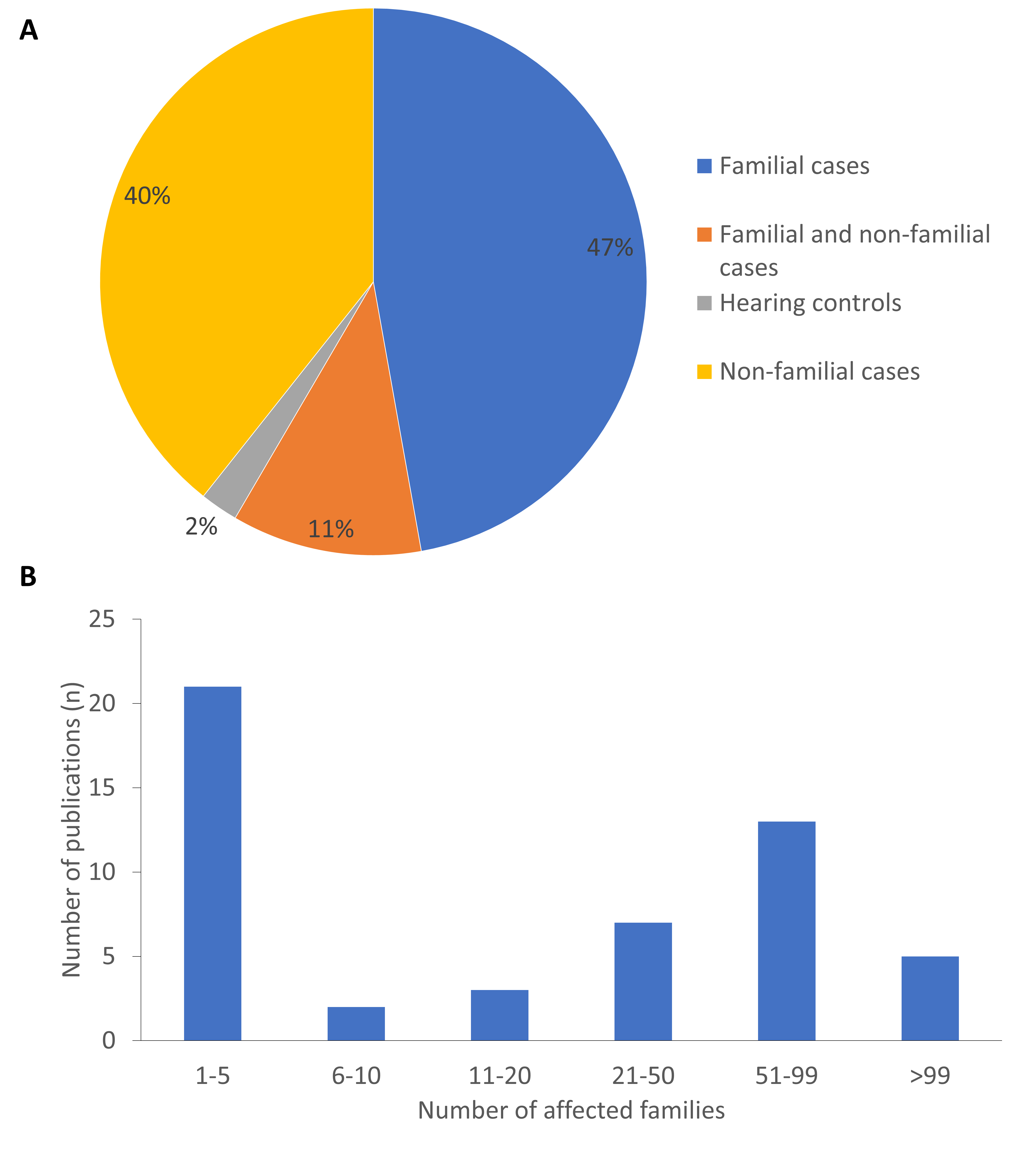


Figure S2: Classification of the publications based on the affected families studied (A) Categorization of the publications based on familial and non-familial cases. (B) A bar graph of the number of affected families investigated per publication.

Table S1: Molecular methods used to investigate HI genes

| Country | Method | *Frequency (n) |
| --- | --- | --- |
| Algeria | Restriction fragment length polymorphism | 1 |
|  | Targeted exome sequencing | 1 |
|  | Targeted sequencing | 5 |
|  | Whole exome sequencing | 2 |
| Cameroon | OtoSCOPE | 1 |
|  | Targeted sequencing | 9 |
|  | Multiplex PCR | 1 |
|  | Whole exome sequencing | 5 |
| Egypt | Targeted sequencing | 6 |
|  | Single-strand conformation polymorphism | 1 |
|  | Restriction fragment length polymorphism | 2 |
| Ghana | Targeted sequencing | 4 |
|  | Multiplex PCR | 1 |
|  | Whole exome sequencing | 1 |
| Kenya | Targeted sequencing | 1 |
| Mauritania | Targeted sequencing | 1 |
| Morocco | Denaturing High Pressure Liquid Chromatography | 1 |
|  | Exome Sequencing | 1 |
|  | Targeted sequencing | 11 |
|  | Restriction fragment length polymorphism | 1 |
|  | Denaturing gradient gel electrophoresis | 1 |
|  | Whole exome sequencing | 4 |
| Nigeria | MiamiOtoGenes | 1 |
|  | Targeted sequencing | 1 |
| São Tomé and Príncipe | Targeted sequencing | 1 |
| Somalia | Targeted sequencing | 1 |
| South Africa | MiamiOtoGenes | 1 |
|  | Karyotyping | 1 |
|  | OtoSCOPE | 2 |
|  | SNaPshot Multiplex System | 1 |
|  | SNP genotyping | 1 |
|  | SureSelect custom kit | 1 |
|  | Targeted sequencing | 3 |
|  | Whole exome sequencing | 1 |
| Sudan | Targeted sequencing | 1 |
| Tunisia | North African deafness chip | 2 |
|  | MiamiOtoGenes | 2 |
|  | Denaturing gradient gel electrophoresis | 1 |
|  | GenScan | 2 |
|  | HaloPlex | 1 |
|  | SNaPshot Multiplex System | 1 |
|  | Targeted sequencing | 22 |
|  | Restriction fragment length polymorphism | 3 |
| Uganda | Targeted sequencing | 1 |

*Frequency = number of times a specific method was used to investigate HI gene variants. Multiple methods were used by 19/89 studies.

Table S2: Clinical significance of pathogenic and likely pathogenic *GJB2* variants reported in Africa

| **Protein change** | **Nucleotide change** | **rs number** | **ClinVar** | **Intervar** | **Varsome** | **Verdict** |
| --- | --- | --- | --- | --- | --- | --- |
| p.(G12Vfs*2) | c.35delG | rs80338939 | Pathogenic | - | Pathogenic | Pathogenic |
| p.(R143W) | c.427C>T | rs80338948 | Pathogenic | Likely Pathogenic | Pathogenic | Pathogenic |
| p.(E47*) | c.139G>T | rs104894398 | Pathogenic | Likely Pathogenic | Pathogenic | Pathogenic |
| p.(V37I) | c.109G>A | rs72474224 | Pathogenic | Likely Pathogenic | Pathogenic | Pathogenic |
| p.(W24*) | c.71G>A | rs104894396 | Pathogenic | Uncertain Significance | Pathogenic | Pathogenic |
| p.(R32C) | c.94C>T | [rs371024165](https://varsome.com/variant/hg19/rs371024165) | Pathogenic | Likely pathogenic | Pathogenic | Pathogenic |
| p.L56Rfs | c.167delT | rs80338942 | Pathogenic |  | Pathogenic | Pathogenic |
| p.(K112Efs*2) | c.334_335 delAA | [rs756484720](https://varsome.com/variant/hg19/rs756484720) | Pathogenic |  | Pathogenic | Pathogenic |
| p.(V178A) | c.533T>C | [rs568612627](https://varsome.com/variant/hg19/rs568612627) | Likely Pathogenic | Likely Pathogenic | Likely Pathogenic | Pathogenic |
| p.(Y142*) | c.425_426del TCinsAA | - | - | - | Pathogenic | Likely Pathogenic |
| p.(W77*) | c.231G>A | [rs80338944](https://varsome.com/variant/hg19/rs80338944) | Pathogenic | Uncertain Significance | Pathogenic | Pathogenic |
| p.(R143Q) | c.428G>A | rs104894401 | Pathogenic | Likely Pathogenic | Pathogenic | Pathogenic |
| p.(L79Cfs ) | c.235delC | [rs80338943](https://varsome.com/variant/hg19/rs80338943) | Pathogenic |  | Pathogenic | Pathogenic |
| p.(W44*) | c.132G>A | [rs104894407](https://varsome.com/variant/hg19/rs104894407) | Pathogenic | Uncertain Significance | Pathogenic | Pathogenic |
| p.(M34T) | c.101T>C | rs35887622 | Pathogenic | Benign | Pathogenic | Pathogenic |
| p.(G200R) | c.598G>A | [rs786204597](https://varsome.com/variant/hg19/rs786204597) | Likely Pathogenic | Uncertain Significance | Likely pathogenic | Pathogenic |
| p.(I203K) | [c.608_609del CinsAA](https://varsome.com/variant/hg19/NM_004004.6:c.608_609delTCinsAA) | - | - | - | Pathogenic | Likely Pathogenic |
| p.(Y136Tfs*32) | c.405delC | - | - | - | Pathogenic | Likely Pathogenic |
| p.(V13Cfs*35) | c.35dupG | rs80338939 | Pathogenic | - | Pathogenic | Pathogenic |
| p.(L79P) | c.236T>C | [rs1555341957](https://varsome.com/variant/hg19/rs1555341957) | Likely Pathogenic | Uncertain Significance | Likely Pathogenic | Pathogenic |
| p.(N206S) | c.617A>G | rs111033294 | Pathogenic | Likely Pathogenic | Pathogenic | Pathogenic |
| p.(E129* ) | c.385G>T | rs397516875 | Pathogenic | Uncertain Significance | Pathogenic | Pathogenic |
| p.(R75Q ) | c.224G>A | [rs28931593](https://varsome.com/variant/hg19/rs28931593) | Pathogenic | Likely Pathogenic | Pathogenic | Pathogenic |
| p.(del120E) | c.358_360del GAG | rs80338947 | Pathogenic |  | Pathogenic | Pathogenic |
| p.(R184P) | c.551G>C | [rs80338950](https://varsome.com/variant/hg19/rs80338950) | Conflicting Interpretations | Likely Pathogenic | Pathogenic | Pathogenic |
| p.(G130A) | c.389G>C | rs779018464 | - | Likely Pathogenic | Pathogenic | Pathogenic |
| p.(R81N) | c.241_243del CTGinsAAC | - | - | - | Pathogenic | Likely Pathogenic |
| p.(I20Mfs*15) | [c.60delT](https://varsome.com/variant/hg19/NM_004004.6:c.60delT) | - | - | - | Pathogenic | Likely Pathogenic |

Table S3: Pathogenic and Likely Pathogenic *GJB2* variants reported in Africa.

| **Reference** | **Year of publication** | **Country** | **c.35delG: p.(G12Vfs*2)** | **c.427C>T: p.(R143W)** | **c.139G>T:**  **p.(E47*)** | **c.109G>A:**  **p.(V37I)** | **c.71G>A:**  **p.(W24*)** | **c.94C>T:**  **p.(R32C)** | **c.167delT: p.(L56Rfs)** |
| --- | --- | --- | --- | --- | --- | --- | --- | --- | --- |
| Abidi | 2007 | Morocco | 61/162 | - | 1/162 | 3/162 | - | - | - |
| Adadey | 2019 | Ghana | - | 64/442 | - | - | - | - | - |
| Adadey | 2020 | Ghana | - | 15/36 | - | - | - | - | - |
| Ammar-Khodja | 2015 | Algeria | 55/130 | - | 7/130 | - | - | - | - |
| Ammar-Khodja | 2009 | Algeria | 36/118 | - | 1/118 | 3/118 | - | - | 2/118 |
| Ammar-Khodja | 2007 | Algeria | 27/116 | - | - | - | - | - | - |
| Bakhchane | 2016 | Morocco | 107/304 | - | 1/304 | 10/304 | - | - | - |
| Belguith | 2009 | Tunisia | 44/204 | - | - | - | - | - | - |
| Arab | 2000 | Tunisia | 6/14 | - | 2/14 | - | - | - | - |
| Ben Said | 2012 | Tunisia | - | - | 10/50 | - | - | - | - |
| Brobby | 1998 | Ghana | - | 22/22 | - | - | - | - | - |
| El Barbary | 2013 | Egypt | 11/102 | - | - | - | - | - | - |
| El Barbary | 2015 | Egypt | 11/102 | - | - | - | - | - | - |
| Elbagoury | 2014 | Egypt | 13/72 | - | - | - | - | - | - |
| Gazzaz | 2005 | Morocco | 50/232 | - | - | - | - | - | - |
| Gibriel | 2019 | Egypt | - | - | - | - | 14/206 | - | - |
| Hamelmann | 2001 | Ghana | - | 110/730 | - | - | - | - | - |
| Masmoudi | 2000 | Tunisia | 20/140 | - | 2 | - | - | - | - |
| Moctar | 2016 | Mauritania | 5/278 | - | - | - | - | 4/278 | - |
| Mohamed | 2010 | Egypt | 27/310 | - | - | - | - | - | - |
| Riahi Chahed | 2013 | Tunisia | 2/4 | - | - | - | - | - | - |
| Riahi Hammami | 2013 | Tunisia | 82/262 | - | 8/262 | 2/262 | - | - | - |
| Riahi Zainine | 2013 | Tunisia | - | - | - | 1/2 | - | - | - |
| Snoeckx Hassan | 2005 | Egypt | 24/222 | - | - | 3/222 | - | - | - |
| MoctarEly Cheikh | 2016 | Morocco | 13/278 | - | - | - | - | 10/278 | - |
| Ratbi | 2007 | Morocco | 24/50 | - | - | - | - | - | - |
| Talbi Crystel Bonnet | 2019 | Algeria | 36/182 | - | 4/182 | - | - | - | 2/182 |
| Talbi | 2018 | Algeria | 8/22 | - | - | - | - | - | - |
| Trabelsi, | 2013 | Tunisia | 45/190 | - | 3/190 | 1/190 | - | - | - |
|  | | | | | | | | | |
| **Reference** | **Year of publication** | **Country** | **c.334_335delAA: p.(K112Efs*2)** | **c.533T>C: p.(V178A)** | **c.425_426delTC insAA: p.(Y142*)** | **c.231G>A: p.(W77*)** | **c.428G>A: p.(R143Q)** | **c.235delC: p.(L79Cfs)** | **c.132G>A: p.(W44*)** |
| Adadey | 2019 | Ghana | - | - | - | - | - | - | 2/442 |
| Bosch Jean Jacques | 2014 | Cameroon | - | - | 1/360 | - | - | - | - |
| Hamelmann | 2001 | Ghana | - | 4/730 | - | - | - | - | - |
| Javidnia, | 2014 | Uganda | - | - | - | 3/252 | - | - | - |
| Riahi Hammami | 2013 | Tunisia | - | - | - | - | - | 2/262 | - |
| Riahi Zainine | 2013 | Tunisia | - | - | - | - | 1/2 | - | - |
| Snoeckx Hassan | 2005 | Egypt | 2/222 | - | 1/222 | - | - | - | - |
| Trabelsi, | 2013 | Tunisia | 2/190 | - | - | - | - | - | - |
|  | | | | | | | | | |
| **Reference** | **Year of publication** | **Country** | **c.101T>C: p.(M34T)** | **c.598G>A: p.(G200R)** | [**c.608_609del TCinsAA: p.(I203K)**](https://varsome.com/variant/hg19/NM_004004.6:c.608_609delTCinsAA) | **c.405delC p.(Y136Tfs*32)** | **c.35dupG: p.V13Cfs*35** | **c.236T>C: p.(L79P)** | **c.617A>G: p.(N206S)** |
| Ammar-Khodja | 2009 | Algeria | - | - | - | - | - | - | 1/118 |
| Bakhchane | 2016 | Morocco | - | 2/304 | - | - | - | - | - |
| Caroca | 2016 | São Tomé and Príncipe | 2/272 | - | - | - | - | - | - |
| Hamelmann | 2001 | Ghana | - | - | 2/730 | - | 1/730 | 1/730 | - |
| Riahi Chahed | 2013 | Tunisia | - | - | - | 2/4 | - | - | - |
|  | | | | | | | | | |
| **Reference** | **Year of publication** | **Country** | **c.385G>T: p.(E129*)** | **c.224G>A: p.(R75Q)** | **c.358_360 delGAG: p.(del120E)** | **c.551G>C: p.(R184P)** | **c.389G>C: p.(G130A)** | **c.241_243del CTGinsAAC: p.(R81N)** | [**c.60delT: p.I20Mfs*15**](https://varsome.com/variant/hg19/NM_004004.6:c.60delT) |
| Bakhchane | 2016 | Morocco | 1/304 | 1/304 | 1/304 | - | - |  | - |
| Gibriel | 2019 | Egypt | - | - | - | - | - | 1/206 | - |
| Hamelmann | 2001 | Ghana | - | - | - | 1/730 | - | - | - |
| Riahi Hammami | 2013 | Tunisia | - | - | - | - | 1/262 | - | - |
| Meguid | 2013 | Egypt | - | - | - | - | - | - | 1/188 |

The numerators in this table represent the number of mutated alleles, and the denominators the total number of screened alleles.

Table S4: *GJA1* and *GJB4* variants reported in Africa

| Country | ^#^Allele frequency | Gene | Protein | Coding | rs number | InterVar | VarSome | Reference |
| --- | --- | --- | --- | --- | --- | --- | --- | --- |
| Cameroon | 2/150 | *GJA1-Cx43* | - | c.-67A>G | rs189167598 | - | Benign | (Bosch et al. 2014a) |
| South Africa | 1/50 | *GJA1-Cx43* | p.(N63N) | c.189T>C | rs139688042 | Likely benign | Uncertain Significance |  |
| South Africa | 2/50 | *GJA1-Cx43* | p.(N122N) | c.366T>C | - | Likely benign | Uncertain Significance |  |
| Cameroon | 11/150 | *GJA1-Cx43* | p.(R239R) | c.717G>A | rs57946868 | Benign | Uncertain Significance |  |
| South Africa | 2/50 | *GJA1-Cx43* |  |  |  |  |  |  |
| South Africa | 1/50 | *GJA1-Cx43* | p.(A253V) | c.758C>T | rs17653265 | Benign | Benign |  |
| Ghana | 2/400 | *GJB4-Cx30.3* | p.(N119T) | c.356A>C | rs190460237 | Likely Pathogenic | Uncertain Significance | (Adadey et al. 2020a) |
| Ghana | 152/400 | *GJB4-Cx30.3* | p.(E204A) | 611A>C | rs3738346 | Benign | Benign |  |
| Ghana | 127/400 | *GJB4-Cx30.3* | p.(R151S) | c.451C>A | rs78499418 | Benign | Benign |  |
| Ghana | 30/400 | *GJB4-Cx30.3* | p.(T172T) | c.516T>C | rs111693060 | Benign | Benign |  |
| Ghana | 5/400 | *GJB4-Cx30.3* | p.(K123K) | c.369G>A | rs142843509 | Likely Benign | Benign |  |
| Ghana | 31/400 | *GJB4-Cx30.3* | p.(R101R) | c.303C>G | rs138184343 | Likely Benign | Benign |  |
| Ghana | 8/400 | *GJB4-Cx30.3* | p.(Q80*) | c.238C>T | rs114429815 | Benign | Benign |  |

^#^Allele frequency (the numerators in this column represent the number of mutated alleles, and the denominators are the total number of screened alleles), InterVar, and VarSome are databases to assess the clinical significance of the variants.

Table S5: Non-*GJB2* hearing impairment genes identified in Africa

| Gene | Variant | rs-number | ^#^Allele frequency | VarSome | InterVar | ClinVar | Country | Reference |
| --- | --- | --- | --- | --- | --- | --- | --- | --- |
| *CDH23* | c.6399C>A: p.(D2133E) | - | 1/20 | Uncertain Significance | Uncertain Significance | - | Cameroon | (Lebeko et al. 2016) |
| *CDH23* | c.8720T>C: p.(M2907T) | - | 1/20 | Uncertain Significance | Uncertain Significance | - | Cameroon | (Lebeko et al. 2016) |
| *CIB2* | c.310C > T: p.(R104*) | rs1054728914 | 2/44 | Pathogenic | - | - | Tunisia | (Souissi et al. 2021) |
| *CIB2* | c.97C > T: p.(R33*) | [rs201845656](https://varsome.com/variant/hg19/rs201845656?&annotation-mode=germline) | 4/22 | Pathogenic | pathogenic | Pathogenic | Algeria | (Talbi et al. 2018) |
| *CLIC5* | c.224T>C; p.(L75P) | - | 2/2 | Pathogenic | Pathogenic | - | Cameroon | (Wonkam-Tingang et al. 2020) |
| *CLIC5* | c.63+1G>A | - | 2/2 | Pathogenic | Pathogenic | - | Cameroon | (Wonkam-Tingang et al. 2020) |
| *COL9A3* | c.G406A: p.(G136S) | rs145035835 | 2/36 | Likely benign | Uncertain Significance | Likely benign | Cameroon | (Wonkam et al. 2021b) |
| *DXML2* | c.918G>T; p.(Q306H) | - | 2/2 | Uncertain Significance | Uncertain Significance | - | Cameroon | (Wonkam-Tingang et al. 2021) |
| *ESRRB* | c.884A > G: p(Y295C) | rs780275423 | 2/44 | Uncertain Significance | Uncertain Significance | - | Tunisia | (Souissi et al. 2021) |
| *GRXCR2* | c.251delC: p.(R84fs) | - | 2/8 | - | - | - | Cameroon | (Wonkam et al. 2021a) |
| *LHFPL5* | c.89dupG p.(T31Yfs*41) | [rs756030149](https://varsome.com/variant/hg19/rs756030149?&annotation-mode=germline) | 2/258 | Pathogenic | - | Likely Pathogenic | Tunisia | (Bensaid et al. 2011) |
| *LOXHD1* | c.3371G>A: p.(R1124H) | rs762111513 | 1/20 | Uncertain Significance | Uncertain Significance | - | Cameroon | (Lebeko et al. 2016) |
| *LOXHD1* | c.3979T>A: p.(F1327I) | rs749667545 | 1/20 | Uncertain Significance | Uncertain Significance | - | Cameroon | (Lebeko et al. 2016) |
| *LRIG1* (candidate) | c.1657G>A: p.(G553R) | rs77775448 | 1/36 | Uncertain Significance | Uncertain Significance | - | Cameroon | (Oluwole et al. 2021) |
| *LRTOMT* | c.242G>A: p.(R81Q) | [rs137853185](https://varsome.com/variant/hg19/rs137853185?&annotation-mode=germline) | 4/76 | Likely Pathogenic | Likely Pathogenic | Pathogenic | Tunisia | (Yan et al. 2016a) |
| *MARVELD2* | c.1555−1G>A | - | 2/182 | Pathogenic | - | - | South Africa | (Yan et al. 2016a) |
| *MCPH1* (candidate) | c.2311C>G p.(P771A) | [rs369802722](https://varsome.com/variant/hg19/rs369802722?&annotation-mode=germline) | 2/36 | Likely benign | Uncertain Significance | - | Cameroon | (Oluwole et al. 2021) |
| *MPZL2* | c.72delA: p.(I24MfsTer22) |  | 2/2 | Pathogenic | - | Pathogenic | Morocco | (Amalou et al. 2021) |
| *MYO15A* | c.4888 T: p.(R1630C) | rs138861831 | 2/36 | Uncertain Significance | Uncertain Significance | Conflicting Interpretations | Cameroon | (Wonkam et al. 2021b) |
| *MYO15A* | c.4998C>A: p.(C1666*) | - | 2/132 | Pathogenic | Pathogenic | - | Tunisia | (Belguith et al. 2009) |
| *MYO15A* | c.5417T>C: p.(L1806P) | - | 2/76 | Uncertain Significance | Uncertain Significance | - | Tunisia | (Yan et al. 2016a) |
| *MYO15A* | c.6265 T > C: p.(F2089L) | - | 2/44 | Likely Pathogenic | Uncertain Significance | - | Tunisia | (Souissi et al. 2021) |
| *MYO15A* | c.7395+3G>A | [rs748492850](https://varsome.com/variant/hg19/rs748492850?&annotation-mode=germline) | 37/132 | Uncertain Significance | - | - | Tunisia | (Belguith et al. 2009) |
| *MYO15A* | c.7395+3G>C | [rs748492850](https://varsome.com/variant/hg19/rs748492850?&annotation-mode=germline) | 2/76 | Uncertain Significance | - | - | Tunisia | (Yan et al. 2016a) |
| *MYO15A* | c.9229+1G>C | - | 55/132 | Pathogenic | - | - | Tunisia | (Belguith et al. 2009) |
| *MYO3A* | c.C424T: p.(H142Y) | rs140301218 | 2/36 | Likely benign | Likely benign | Likely benign | Cameroon | (Wonkam et al. 2021b) |
| *MYO6* | c.1477_1487delCAAGAACTCTA: p.(Q493Sfs*8) | - | 2/180 | Pathogenic | - | - | Nigeria | (Yan et al. 2016a) |
| *MYO7A* | c.1334T>G: p.(Y445S) | - | 2/96 | Likely Pathogenic | Uncertain Significance | | Tunisia | (Chakchouk et al. 2015) |
| *MYO7A* | c.1708C>T: p.(R570*) | [rs1591310948](https://varsome.com/variant/hg19/rs1591310948?&annotation-mode=germline) | 1/180 | Pathogenic | Pathogenic | Pathogenic | Nigeria | (Yan et al. 2016a) |
| *MYO7A* | c.1996C>T: p.(R666) | - | 2/20 | Likely benign | Likely benign | - | Cameroon | (Lebeko et al. 2016) |
| *MYO7A* | c.1996C>T: p.(R666) | - | 5/114 | Likely benign | Likely benign | - | Cameroon | (Lebeko et al. 2017) |
| *MYO7A* | c.287C>T: p.(T96M) | [rs781811444](https://varsome.com/variant/hg19/rs781811444?&annotation-mode=germline) | 1/180 | Pathogenic | Likely Pathogenic | Likely Pathogenic | Nigeria | (Yan et al. 2016a) |
| *MYO7A* | c.470+1G > A | [rs797044510](https://varsome.com/variant/hg19/rs797044510?&annotation-mode=germline) | 4/22 | Pathogenic | - | Pathogenic | Algeria | (Talbi et al. 2018) |
| *MYO7A* | c.5806_5808 delCTC: p.(L1937*) | - | 1/20 | Pathogenic | - |  | Cameroon | (Lebeko et al. 2016) |
| *OCM2* (candidate) | c.227G>C: p.(R76T) | [rs139724916](https://varsome.com/variant/hg19/rs139724916?&annotation-mode=germline) | 1/36 | Uncertain Significance | Uncertain Significance | - | Cameroon | (Oluwole et al. 2021) |
| *OTOF* | c.169G > T: p.(E57*) | rs397515591 | 2/44 | Pathogenic | Pathogenic | - | Tunisia | (Souissi et al. 2021) |
| *OTOF* | c.766-2A>G | [rs80356584](https://varsome.com/variant/hg19/rs80356584?&annotation-mode=germline) | 2/8 | Pathogenic | - | Pathogenic | Cameroon | (Wonkam et al. 2021a) |
| *OTOF* | c.766-2A>G | rs80356584 | 2/20 | Pathogenic | - | Pathogenic | Cameroon | (Lebeko et al. 2016) |
| *OTOF* | c.766-2A>G | rs80356584 | 3/114 | Pathogenic | - | Pathogenic | Cameroon | (Lebeko et al. 2017) |
| *PJVK* | c.113_114insT: p.(K41Gfs*8) | [rs1559365985](https://varsome.com/variant/hg19/rs1559365985?&annotation-mode=germline) | 2/2 | Pathogenic | - | - | Morocco | (Ebermann et al. 2007) |
| *POU3F4* | c.986G>C: p.(R329P) | - | 2/182 | uncertain Significance | Uncertain Significance |  | South Africa | (Yan et al. 2016a) |
| *SIX1* | c.373G>A: p.(E125K) | [rs797044960](https://varsome.com/variant/hg19/rs797044960?&annotation-mode=germline) | 1/182 | Likely Pathogenic | Likely Pathogenic | Pathogenic | South Africa | (Yan et al. 2016a) |
| *SLC12A2* | c.2935G>A: p.(E979K) | - | 2/2 | Likely Pathogenic | Likely Pathogenic |  | Ghana | (Adadey et al. 2021) |
| *SLC22A4* | c.338G>A: p.(C113Y) | rs768484124 | 2/2 | Likely Pathogenic | Uncertain Significance | - | Morocco | (Chiereghin et al. 2021) |
| *SLC22A4* | c.338G>A: p.(C113Y) | rs768484124 | 2/44 | Uncertain Significance | Uncertain Significance | - | Tunisia | (Souissi et al. 2021) |
| *SLC26A4* | c.164+1G>C | [rs1021962985](https://varsome.com/variant/hg19/rs1021962985?&annotation-mode=germline) | 1/180 | Pathogenic | - | - | Nigeria | (Yan et al. 2016a) |
| *SLC26A4* | c.1678G>A: p.(D560N) | [rs759360026](https://varsome.com/variant/hg19/rs759360026?&annotation-mode=germline) | 2/8 | Likely Pathogenic | Uncertain Significance | Uncertain Significance | Cameroon | (Wonkam et al. 2021a) |
| *SLC26A4* | c.1678G>A: p.(D560N) | rs759360026 | 1/20 | Likely Pathogenic | Uncertain Significance | Uncertain Significance | Cameroon | (Lebeko et al. 2016) |
| *SLC26A4* | c.2007C>A: p.(D669E) | - | 1/20 | Pathogenic | Likely Pathogenic | - | Cameroon | (Lebeko et al. 2016) |
| *SLC26A4* | c.2171A>T: p.(D724V) | - | 1/180 | Likely Pathogenic | Likely Pathogenic | - | Nigeria | (Yan et al. 2016a) |
| *SLC26A4* | c.251delC p.(I85Sfs*33) | - | 2/8 | Pathogenic | - | - | Cameroon | (Wonkam et al. 2021a) |
| *SLC26A4* | c.410C > T:p.(S137L) | - | 4/22 | Uncertain Significance | Uncertain Significance | | Algeria | (Talbi et al. 2018) |
| *SLC26A4* | c.737delA: p.(N246Tfs*43) | [rs918684449](https://varsome.com/variant/hg19/rs918684449?&annotation-mode=germline) | 2/180 | Pathogenic | - | Likely Pathogenic | Nigeria | (Yan et al. 2016a) |
| *SPNS2* | c.867C>A p.(P289Q) | - | 1/36 | Uncertain Significance | Uncertain Significance | - | Cameroon | (Oluwole et al. 2021) |
| *STRC* | 20-kb del (CNV) | - | 1/20 | - | - | - | Cameroon | (Lebeko et al. 2016) |
| *TBC1D24* | c.457G>A: p.(E153K) | rs376712059 | 2/4 | Likely Pathogenic | Uncertain Significance | Conflicting Interpretations | Moroco | (Bakhchane et al. 2015a) |
| *TBC1D24* (candidate) | c.641G>A: p.(R214H) | rs200324356 | 2/4 | Likely Pathogenic | Uncertain Significance | Conflicting Interpretations | Moroco | (Bakhchane et al. 2015a)t |
| *TECTA* | c.2743A > G: p.(I15V) | - | 2/22 | Pathogenic | - | - | Algeria | (Talbi et al. 2018) |
| *TECTA* | c.5272+1G > A | - | 2/4 | Pathogenic | - | - | Algeria | (Behlouli et al. 2016) |
| *TMC1* | c.100C>T p.(R34*) | [rs121908073](https://varsome.com/variant/hg19/rs121908073?&annotation-mode=germline) | 10/280 | Pathogenic | Pathogenic | Pathogenic | Tunisia | (Tlili et al. 2008) |
| *TMC1* | c.100C>T p.(R34*) | [rs121908073](https://varsome.com/variant/hg19/rs121908073?&annotation-mode=germline) | 2/22 | Pathogenic | Pathogenic | Pathogenic | Tunisia | (Tlili et al. 2008) |
| *TMC1* | c.1165C>T p.(R389*) | [rs151001642](https://varsome.com/variant/hg19/rs151001642?&annotation-mode=germline) | 2/280 | Pathogenic | Likely Pathogenic | | Tunisia | (Tlili et al. 2008) |
| *TMC1* | c.1764G>A p.(W588*) | [rs368084452](https://varsome.com/variant/hg19/rs368084452?&annotation-mode=germline) | 2/280 | Pathogenic | Pathogenic | - | Tunisia | (Tlili et al. 2008) |
| *TMC1* | c.1810C>G p.(R604G) | - | 2/2 | Uncertain Significance | Uncertain Significance | - | Morocco | (Bakhchane et al. 2015b) |
| *TMPRSS3* | 753 G>C p.(W251C) | [rs137852999](https://varsome.com/variant/hg19/rs137852999?&annotation-mode=germline) | 2/4 | Likely Pathogenic | Pathogenic | Likely Pathogenic | Tunisia | (Masmoudi et al. 2001) |
| *TMPRSS3* | c.1221 C>T: p.(P404L) | [rs28939084](http://www.ncbi.nlm.nih.gov/snp/?term=rs28939084) | 2/4 | Likely Pathogenic | Likely Pathogenic | Pathogenic | Tunisia | (Masmoudi et al. 2001) |
| *TRIOBP* | c.3510_3513dupTGCA: p.(P1172Cfs*13) | - | 1/182 | Pathogenic | - | - | South Africa | (Yan et al. 2016a) |
| *TRIOBP* | c.572delC: p.(P191Rfs*50) | - | 1/182 | Pathogenic | - | - | South Africa | (Yan et al. 2016a) |
| *WHRN* | c.2423delG: p.(G808Afs*11) | [rs869320674](https://varsome.com/variant/hg19/rs869320674?&annotation-mode=germline) | 2/124 | Pathogenic | - | Pathogenic | Tunisia | (Tlili et al. 2005) |
| *WHRN* | c.2423delG: p.(G808Afs*11) | [rs869320674](https://varsome.com/variant/hg19/rs869320674?&annotation-mode=germline) | 2/44 | Pathogenic | - | Pathogenic | Tunisia | (Souissi et al. 2021) |

^#^Allele frequency (the numerators in this column represent the number of mutated alleles, and the denominators are the total number of screened alleles), Multiple affected people in the same family are considered as one unit. InterVar, VarSome, and ClinVar are databases to assess the clinical significance of the variants.

Table S6: PLP mitochondrial variants reported in Africa

| Variant | Allele frequency | Country | Reference |
| --- | --- | --- | --- |
| MTRNR1: 1048C>T | 2/140 | Cameroon | (Souissi et al. 2021; Trotta et al. 2011) |
| MTRNR1: 1462G>T | 2/140 | Cameroon |  |
| MTRNR1: 1018G>A | 2/140 | Cameroon |  |
| MTRNR1: 1503G>A | 4/140 | Cameroon |  |
| m.1555A>G | 2/200 | Tunisia | (Mkaouar-Rebai et al. 2006) |
| m.1555A>G | 2/196 | Tunisia | (Bardien et al. 2009) |
| m.1555A>G | 6/328 | Morocco | (Nahili et al. 2010) |
| m.1555A>G | 4/400 | Tunisia | (Mkaouar-Rebai et al. 2013b) |
| m.735A>G | 4/400 | Tunisia |  |
| m.735A>G | 2/2 | Tunisia | (Mkaouar-Rebai et al. 2010) |
| m.7444G>A | 2/2 | Tunisia | (Mkaouar-Rebai et al. 2013a) |
| m.6498C>A: p.(L199I) | 2/2 | Tunisia |  |
| m.9267G>C: p.(A21P) | 2/2 | Tunisia | (Tabebi et al. 2015) |
| m.5913G>A: p.(D4N) | 2/2 | Tunisia |  |

^#^Allele frequency (the numerators in this column represent the number of mutated alleles, and the denominators are the total number of screened alleles) Multiple affected people in the same family are considered as one unit.

Table S7: Syndromic hearing impairment gene variants from Africa

| Gene | Variant | rs number | ^#^Allele frequency | Syndrome | VarSome | InterVar | ClinVar | Country | Reference |
| --- | --- | --- | --- | --- | --- | --- | --- | --- | --- |
| *ALMS1* | c.10388-2A>G | - | 2/2 | Alström Syndrome | - | - | - | Tunisia | (Ben-Rebeh et al. 2016) |
| *ATP6V1B1* | c.1102G > A: p.(E368K) | - | 4/40 | Distal renal tubular acidosis | Uncertain Significance | - | - | Tunisia | (Elhayek et al. 2013) |
| *ATP6V1B1* | c.1155dupC: p.(I386Hfs*56) | [rs781969081](https://varsome.com/variant/hg19/rs781969081?&annotation-mode=germline) | 6/8 | Distal renal tubular acidosis | Pathogenic | - | Pathogenic | Algeria | (Dahmani et al. 2020) |
| *ATP6V1B1* | c.1155dupC: p.(I386Hfs*56) | [rs781969081](https://varsome.com/variant/hg19/rs781969081?&annotation-mode=germline) | 4/6 | Distal renal tubular acidosis | Pathogenic | - | Pathogenic | Morocco | (Boualla et al. 2016) |
| *ATP6V1B1* | c.1155dupC: p.(I386Hfs*56) | [rs781969081](https://varsome.com/variant/hg19/rs781969081?&annotation-mode=germline) | 24/40 | Distal renal tubular acidosis | Pathogenic | - | Pathogenic | Tunisia | (Elhayek et al. 2013) |
| *ATP6V1B1* | c.1155dupC: p.(I386Hfs*56) | [rs781969081](https://varsome.com/variant/hg19/rs781969081?&annotation-mode=germline) | 23/54 | Distal renal tubular acidosis | Pathogenic | - | Pathogenic | Tunisia | (Nagara et al. 2014) |
| *ATP6V1B1* | c.1169dupC: p.(S391Pfs*51) | - | 2/6 | Distal renal tubular acidosis | Pathogenic | - | - | Morocco | (Boualla et al. 2016) |
| *ATP6V1B1* | c.1221delG: p.(M408Cfs*10) | - | 2/40 | Distal renal tubular acidosis | Pathogenic |  |  | Tunisia | (Elhayek et al. 2013) |
| *ATP6V1B1* | c.175-1G > C | [rs1572919267](https://varsome.com/variant/hg19/rs1572919267?&annotation-mode=germline) | 2/8 | Distal renal tubular acidosis | Pathogenic | - | Pathogenic | Algeria | (Dahmani et al. 2020) |
| *ATP6V1B1* | c.175-1G > C | [rs1572919267](https://varsome.com/variant/hg19/rs1572919267?&annotation-mode=germline) | 6/40 | Distal renal tubular acidosis | Pathogenic | - | Pathogenic | Tunisia | (Elhayek et al. 2013) |
| *ATP6V1B1* | c.175-1G > C | [rs1572919267](https://varsome.com/variant/hg19/rs1572919267?&annotation-mode=germline) | 8/54 | Distal renal tubular acidosis | Pathogenic | - | Pathogenic | Tunisia | (Nagara et al. 2014) |
| *ATP6V1B1* | c.89C>T: p.(T30I) | [rs17720303](https://varsome.com/variant/hg19/rs17720303?&annotation-mode=germline) | 4/54 | Distal renal tubular acidosis | Benign | Benign | Benign | Tunisia | (Nagara et al. 2014) |
| *ERCC8* | c.551-1G>A | rs1554073316 | 2/2 | Cockayne syndrome | Pathogenic | - | - | Somalia | (Kleppa et al. 2007) |
| *GJB2* | c.148G>A, p.(D50N) | rs28931594 | 2/2 | Keratitis-Ichthyosis-Deafness (KID) syndrome | Pathogenic | Likely pathogenic | Pathogenic | Cameroon | (Wonkam et al. 2013) |
| *KCNQ1* | c.1343dupC, p.(E449 Rfs*14) | [rs397508087](https://varsome.com/variant/hg19/rs397508087?&annotation-mode=germline) | 2/2 | Jervell and LangeNielsen syndrome | Pathogenic | Pathogenic | - | Morocco | (Adadi et al. 2017) |
| *MYO7A* | c.1118G>A p.(R373H) | rs201491278 | 1/92 | Usher | Likely pathogenic | Uncertain significance | - | South Africa | (Kabahuma et al. 2021) |
| *MYO7A* | c.1142C>T p.(T381M) | rs782681743 | 1/92 | Usher | Likely pathogenic | Uncertain significance | Uncertain significance | South Africa | (Kabahuma et al. 2021) |
| *MYO7A* | c.1554+7C>T |  | 1/92 | Usher |  |  |  | South Africa | (Kabahuma et al. 2021) |
| *MYO7A* | c.1845delG: p.(K615Nfs*6) | - | 2/8 | Usher | - | - | - | Tunisia | (Ben-Rebeh et al. 2016) |
| *MYO7A* | c.1849T>C: p.(S617P) | rs782063761 | 1/92 | Usher | Likely pathogenic | Uncertain significance | Uncertain significance | South Africa | (Kabahuma et al. 2021) |
| *MYO7A* | c.247C>A p.(R83C) | rs781790246 | 1/92 | Usher | Likely pathogenic | Likely pathogenic | Uncertain significance | South Africa | (Kabahuma et al. 2021) |
| *MYO7A* | c.4388G>A p.(R1463H) | rs558085909 | 1/92 | Usher | Likely pathogenic | Uncertain significance | - | South Africa | (Kabahuma et al. 2021) |
| *MYO7A* | c.470+1G>A | [rs797044510](https://varsome.com/variant/hg19/rs797044510?&annotation-mode=germline) | 2/8 | Usher | Pathogenic | - | Pathogenic | Tunisia | (Ben-Rebeh et al. 2016) |
| *MYO7A* | c.5339A>C p.(Y1780S) | rs1555104196 | 5/92 | Usher | Likely pathogenic | Uncertain significance | Uncertain significance | South Africa | (Kabahuma et al. 2021) |
| *MYO7A* | c.5434G>A: p.(E1812K) | [rs377267777](https://varsome.com/variant/hg19/rs377267777?&annotation-mode=germline) | 2/8 | Usher | Like pathogenic | - | Like pathogenic | Tunisia | (Ben-Rebeh et al. 2016) |
| *MYO7A* | c.6375delC p.(P2126Lfs*5) | - | 1/92 | Usher | Pathogenic | - | - | South Africa | (Kabahuma et al. 2021) |
| *MYO7A* | c.986G>A p.(G329D) | - | 1/92 | Usher | Likely pathogenic | Uncertain significance | - | South Africa | (Kabahuma et al. 2021) |
| *PCDH15* | c.400C>T: p.(R134*) | [rs137853003](https://varsome.com/variant/hg19/rs137853003?&annotation-mode=germline) | 2/8 | Usher | Pathogenic | - | Like pathogenic | Tunisia | (Ben-Rebeh et al. 2016) |
| *SLC26A4* | c.1334T>G: p.(L445W) | [rs111033307](https://varsome.com/variant/hg19/rs111033307?&annotation-mode=germline) | 3/86 | Pendred syndrome | Pathogenic | Pathogenic | Pathogenic | Tunisia | (Chakchouk et al. 2015) |
| *RRM2B* | c.786G>T: p.(N262K) | - | 2/2 | Renal dysfunction, rod‐cone dystrophy, and sensorineural hearing loss | - | - | - | South Africa | (Roberts et al. 2020) |
| *SLC29A3* | c.1088G>A: p.(R363Q) | [rs387907066](https://varsome.com/variant/hg19/rs387907066?&annotation-mode=germline) | 2/10 | H syndrome | Like pathogenic | Pathogenic | - | Tunisia | (Jaouadi et al. 2018) |
| *SLC29A3* | c.42delC; p.(S15Pfs*86) | - | 2/10 | H syndrome | Like pathogenic | - | - | Tunisia | (Jaouadi et al. 2018) |
| *SLC29A3* | c.971C>T: p.(P324L) | [rs758201217](https://varsome.com/variant/hg19/rs758201217?&annotation-mode=germline) | 4/10 | H syndrome | Like pathogenic | Uncertain significance | Likely pathogenic | Tunisia | (Jaouadi et al. 2018) |
| *USH1C* | c.7C>T: p.(R3*) | [rs876657624](https://varsome.com/variant/hg19/rs876657624?&annotation-mode=germline) | 2/8 | Usher | Pathogenic | - | Pathogenic | Tunisia | (Ben-Rebeh et al. 2016) |
| *USH1C* | c.91C>T: p.(R31*) | [rs121908370](https://varsome.com/variant/hg19/rs121908370?&annotation-mode=germline) | 2/96 | Usher | Pathogenic | Pathogenic | Pathogenic | Tunisia | (Chakchouk et al. 2015) |
| *USH1G* | c.393insG | [rs587776546](https://varsome.com/variant/hg19/rs587776546?&annotation-mode=germline) | 2/96 | Usher | Pathogenic | - | Pathogenic | Tunisia | (Chakchouk et al. 2015) |
| *USH2A* | c.14586T>G: p.(Y4862*) | - | 4/76 | Usher syndrome | Pathogenic | Likely pathogenic | - | Tunisia | (Yan et al. 2016a) Takin |
| *WFS1* | c.2425G>A; p.(E809K) | [rs71539673](https://varsome.com/variant/hg19/rs71539673?&annotation-mode=germline) | 6/622 | Syndrome of Neonatal/Infancy-Onset Diabetes, Congenital Sensorineural Deafness, and Congenital Cataracts | Uncertain significance | - | Pathogenic | Morocco | (De Franco et al. 2017) |
| *WFS1* | c.2489A>C p.(E830A) | - | 2/622 | Syndrome of Neonatal/Infancy-Onset Diabetes, Congenital Sensorineural Deafness, and Congenital Cataracts | Uncertain significance | - | - | Morocco | (De Franco et al. 2017) |
| *WFS1* | c.937C>T ; p.(H313T) | [rs886044563](https://varsome.com/variant/hg19/rs886044563?&annotation-mode=germline) | 2/622 | Syndrome of Neonatal/Infancy-Onset Diabetes, Congenital Sensorineural Deafness, and Congenital Cataracts | Uncertain significance | Uncertain significance | Like pathogenic | Morocco | (De Franco et al. 2017) |

^#^Allele frequency (the numerators in this column represent the number of mutated alleles, and the denominators are the total number of screened alleles), Multiple affected people in the same family are considered as one unit. InterVar, VarSome, and ClinVar are databases to assess the clinical significance of the variants.
